# WNT signaling enhances breast cancer cell motility and blockade of the WNT pathway by sFRP1 suppresses MDA-MB-231 xenograft growth

**DOI:** 10.1186/bcr2317

**Published:** 2009-05-27

**Authors:** Yutaka Matsuda, Thomas Schlange, Edward J Oakeley, Anne Boulay, Nancy E Hynes

**Affiliations:** 1 Friedrich Miescher Institute for Biomedical Research (Part of the Novartis Research Foundation), Maulbeerstrasse 66, CH-4058 Basel, Switzerland

## Abstract

**Introduction:**

In breast cancer, deregulation of the WNT signaling pathway occurs by autocrine mechanisms. WNT ligands and Frizzled receptors are coexpressed in primary breast tumors and cancer cell lines. Moreover, many breast tumors show hypermethylation of the secreted Frizzled-related protein 1 (sFRP1) promoter region, causing low expression of this WNT antagonist. We have previously shown that the WNT pathway influences proliferation of breast cancer cell lines via activation of canonical signaling and epidermal growth factor receptor transactivation, and that interference with WNT signaling reduces proliferation. Here we examine the role of WNT signaling in breast tumor cell migration and on xenograft outgrowth.

**Methods:**

The breast cancer cell line MDA-MB-231 was used to study WNT signaling. We examined the effects of activating or blocking the WNT pathway on cell motility by treatment with WNT ligands or by ectopic sFPR1 expression, respectively. The ability of sFRP1-expressing MDA-MB-231 cells to grow as xenografts was also tested. Microarray analyses were carried out to identify targets with roles in MDA-MB-231/sFRP1 tumor growth inhibition.

**Results:**

We show that WNT stimulates the migratory ability of MDA-MB-231 cells. Furthermore, ectopic expression of sFRP1 in MDA-MB-231 cells blocks canonical WNT signaling and decreases their migratory potential. Moreover, the ability of MDA-MB-231/sFRP1-expressing cells to grow as xenografts in mammary glands and to form lung metastases is dramatically impaired. Microarray analyses led to the identification of two genes, *CCND1 *and *CDKN1A*, whose expression level is selectively altered *in vivo *in sFRP1-expressing tumors. The encoded proteins cyclin D1 and p21^Cip1 ^were downregulated and upregulated, respectively, in sFRP1-expressing tumors, suggesting that they are downstream mediators of WNT signaling.

**Conclusions:**

Our results show that the WNT pathway influences multiple biological properties of MDA-MB-231 breast cancer cells. WNT stimulates tumor cell motility; conversely sFRP1-mediated WNT pathway blockade reduces motility. Moreover, ectopic sFRP1 expression in MDA-MB-231 cells has a strong negative impact on tumor outgrowth and blocked lung metastases. These results suggest that interference with WNT signaling using sFRP1 to block the ligand- receptor interaction may be a valid therapeutic approach in breast cancer.

## Introduction

The WNT signaling network is complex, with 19 WNT ligands, 10 Frizzled (FZD) receptors, as well as the co-receptors, low-density lipoprotein receptor-related protein (LRP) 5 and LRP6. WNT receptor binding stimulates intracellular signaling, promoting stabilization and nuclear translocation of the key effector of the canonical pathway, β-catenin [1,2]. Intracellular mediators of the WNT pathway are mutated in many human cancers. Inactivating mutations in the *APC *or *AXIN *genes, as well as activating *CTNNB1 *mutations, each causes β-catenin stabilization and nuclear accumulation in the absence of WNT ligands. In the nucleus, β-catenin forms functional complexes with transcription factors of the lymphoid enhancer binding factor-1/T-cell factor (TCF) family, activating expression of target genes with cancer-promoting roles [3]. In addition to activation of the canonical pathway by engagement of FZD and LRP receptors, WNT ligands bind the Ror2 or Ryk receptors to stimulate β-catenin-independent pathways that have been involved with cytoskeletal reorganization and cell migration [2,4].

In breast cancer, deregulation of WNT signaling appears to occur by autocrine mechanisms [5-7]. Multiple WNT ligands and FZD receptors are expressed in primary human breast tumors and in breast cancer cell lines [3,7-9]. Furthermore, most breast tumors show hypermethylation of the promoter region of secreted Frizzled-related protein 1 (sFRP1) and low expression of this negative WNT pathway regulator [10-12]. Interference with autocrine WNT signaling has been shown to block *in vitro *proliferation of many human breast cancer cell lines [6,7]. We have extended these studies and show in the present article that blocking the WNT pathway in MDA-MB-231 breast cancer cells not only decreases proliferation, but also impairs the motility of the tumor cells. Furthermore, we show that stable expression of sFRP1 in MDA-MD-231 cells has a dramatic effect on the ability of the cells to grow as tumor xenografts in nude mice. The results presented here provide further evidence supporting approaches to target WNT pathway activity in breast cancer.

## Materials and methods

### Reagents

The following primary antibodies were used: c-Myc (9E10, to detect Myc-tagged protein), DVL2 and DVL3 (Santa Cruz Biotechnology, Inc., Santa Cruz, CA, USA); DVL1 (R&D Systems, Abingdon, UK); active β-catenin (anti-ABC; Upstate, Billerica, VA, USA); α-tubulin (DM1A) (Neomarkers, Fremont, CA, USA); cyclin D1 (SP4) (Cell MARQUE, Rocklin, CA, USA) for immunohistochemistry (IHC); cyclin D1 (Chemicon, Billerica, MA, USA) for western blotting; bromodeoxyuridine (BrdU) (Roche, Basel Switzerland); p21^Cip1 ^(OP64-100UG) (Oncogene Research Products, Cambridge, MA, USA); ERK and P-ERK (Thr202/Tyr204) (Cell Signaling Technology, Danvers, MA, USA); total β-catenin and CD31 (BD Pharmingen, Franklin Lakes, CA, USA); and active β_1_-integrin (Clone HUTS-4, MAB2079Z; Chemicon).

As secondary antibodies we used anti-rabbit and anti-mouse antibodies (GE Healthcare, Little Chalfont, UK; LI-COR Bioscience, Lincoln, NE, USA), anti-rat antibody (GE Healthcare) or anti-goat antibody (DAKO A/S, Glostrup, Denmark) coupled to horseradish peroxidase (HRP) or IRDye 800CW.

For IHC we used Biotin-SP-conjugated affinipure donkey anti-rabbit, anti-mouse, anti-rat IgG (Jackson ImmunoResearch, West Grove, PA, USA) and goat anti-rat ALEXA 568 (Molecular Probes, Eugene, OR, USA). Recombinant Wnt3a was purchased from R&D Systems. Y27632 was purchased from Sigma-Aldrich (St. Louis, MO, USA). The cDNA encoding Myc/His-tagged human sFRP1 in pCDNA was provided by Jeffrey Rubin (NCI, Bethesda, MD, USA) and was recloned into the pBabePuro retroviral vector. Conditioned media (CM) from Wnt1-producing cells, from sFRP1-producing cells, and purified sFRP1 were prepared as previously described [7].

### T-cell factor reporter assay

MDA-MB-231/sFRP1-P1 cells and control-P1 cells were seeded on 12-well plates and were transfected with a mixture of Super TOPFlash plasmid and pRL-CMV (Promega, Madison, WI, USA) to assay TCF promoter activity using Fugene6 (Roche) according to the manufacturer's instructions. Luciferase activities were measured 48 hours later using the Dual-Luciferase Reporter Assay System (Promega) and Mithras LB940 (Berthold Technologies, Bad Wildbad, Germany) according to the manufacturer's instructions. The fold activation was normalized against renilla luciferase. Nine wells were used per condition and the average and standard error are shown in the graph.

### Cell culture, transfections, retroviral infections, proliferation and anoikis assays

The human breast cancer cell line MDA-MB-231 (ATCC, Manassas, VA, USA) was cultivated in DMEM, 10% heat-inactivated FCS (Amimed, Allschwil, Switzerland) supplemented with penicillin and streptomycin (Sigma-Aldrich). All transfections were performed using FuGENE 6 Transfection Reagent (Roche) following the manufacturer's guidelines. MDA-MB-231 cells were stably transfected with pCDNA3.1(+) (Invitrogen, Carlsbad, CA, USA) encoding Myc/His-tagged human sFRP1 or empty pCDNA3.1(+) as control. After selection with 1 mg/ml G-418, three clones of MDA-MB-231/sFRP1 and three control clones were isolated. Equal cell numbers of these clones were pooled before some experiments (MDA-MB-231/sFRP1-P1 and MDA-MB-231/control-P1). A second pool of sFRP1-expressing MDA-MB-231 cells (MDA-MB-231/sFRP1-P2) and control cells (MDA-MB-231/control-P2), each representing > 100 clones, was generated by infecting the cells with pBabePuro encoding Myc/His-tagged human sFRP1 or empty pBabePuro followed by selection with 2 μg/ml Puromycin (Sigma-Aldrich).

Cell proliferation was measured either by counting cell numbers with a Vi-Cell XR cell viability analyzer (Beckman Coulter, Fullerton, CA, USA) on selected days after seeding 200,000 cells on six-well plates or using the YOPRO cell viability assay (Invitrogen) 3 days after seeding 1,000 cells on a 96-well plate, according to the manufacturer's instructions. Anoikis was measured by seeding cells in 1% FCS-containing medium on polyHema-coated plates to prevent adhesion. Cells were harvested 24 hours later, stained with propidium iodide. The cell cycle distribution was analyzed with a FACScalibur (Becton Dickinson, San Jose, CA, USA) using the Cellquest software. A representative cell cycle distribution of three MDA-MB-231/sFRP1 clones and three control clones is shown. Unless otherwise noted, *P *values were calculated using Student's *t *test.

### Protein extraction and western blotting

Cells were lysed in 1% Nonidet P-40, 50 mM Tris pH 7.5, 120 mM NaCl, 5 mM ethylenediamine tetraacetic acid, 1 mM ethylene glycol tetra-acetic acid (EGTA), 2 mM sodium vanadate, 20 mM β-glycerophosphate, 10 μg/ml aprotinin, 10 μg/ml leupeptin, 0.5 mM phenylmethanesulphonylfluoride (PMSF), 50 mM NaF, 1 mM dithiothreitol for 5 minutes on ice before collecting lysates. Debris was removed by centrifugation at 4°C and the protein concentration was determined using the Bradford reagent (BioRad, Hercules, CA, USA).

For western blotting, protein loading buffer was added to 30 to 50 μg total protein and the samples were denatured for 10 minutes at 95°C prior to separation on SDS-polyacrylamide gels and blotting by semi-dry transfer for 90 minutes on PVDF membranes (Millipore, Billerica, MA, USA). Membranes were blocked using 10% horse serum in Tris-buffered saline- Tween buffer for 1 hour (0.2 M NaCl, 25 mM Tris, pH 7.5, 0.5 ml/l Tween-20), except for p21^Cip ^detection where PBS- Tween buffer was used instead of TBS-Tween buffer for blocking. Blots were incubated with primary antibodies at room temperature for 1 hour or at 4°C overnight, followed by 30-minute incubation with secondary antibodies. These antibodies were anti-rabbit-HRP, anti-mouse-HRP (1:5000) or anti-goat-HRP (1:5000) for detection of luminescence, which was carried out using ECL (GE Healthcare) according to the manufacturer's instructions and using X-OMAT LS films (Kodak, New York, NY, USA). Secondary antibodies IRDye 800CW goat anti-rabbit-IgG or anti-mouse-IgG (1:10,000; LI-COR Biosciences) were detected with the LI-COR Odyssey system according to the manufacturer's instructions (LI-COR Biosciences). Quantification of protein expression was carried out using Odyssey 2.1 (LI-COR Biosciences).

### Wound healing assay

Cells were seeded on six-well plates and grown to confluency. Monolayers were scratched, and in the indicated experiments the media were changed to Wnt1 CM or control CM. When using recombinant Wnt3a and Y27632, 90 minutes before the scratch was made the media were changed to DMEM 10% FBS containing 100 μg/ml Wnt3a (R&D Systems) and/or 5 μM Y27632 (Sigma-Aldrich). Pictures of randomly-chosen nine wound edges per condition were taken at time 0 and at the indicated time points using Nikon DIAPHOT (Nikon, Tokyo, Japan). The recovered area was calculated using ImageQuant TL (GE Healthcare). In some experiments, purified sFRP1 was added to the CM [7].

### Fluorescence-activated cell sorting analysis for active β_1_-integrin

MDA-MB-231/sFRP1-P1 and control-P1 cells (0.3 × 10^6^) were incubated for 30 minutes on ice with an antibody recognizing active β_1_-integrin (final concentration 20 ng/μl) in HEPES/NaCl buffer. This was followed by incubation for 30 minutes on ice with fluorescein isothiocyanate-conjugated (FITC) donkey anti-mouse IgG secondary antibody (Jackson Laboratories) (diluted 1:250) in Flow PBS (1 × PBS, 2% horse serum, 0.1% sodium azide). Fluorescence was measured using a FACSCalibur machine (Becton Dickinson) and the percentage of gated cells stained with active β_1_-integrin was calculated.

### *In vivo *experiments with MDA-MB-231 cells

Female Balb/c nude mice 7 to 10 weeks old were obtained from Charles River Laboratories (L'Arbresle, France) and were maintained in accordance with the Swiss guidelines for animal safety. Mammary tumors were established in mice (5 to 8 mice per group) by injecting 0.5 to 1.0 × 10^6 ^control or sFRP1-expressing MDA-MB-231 cell lines in 100 to 150 μl PBS into the fourth right-side mammary fat pad. The tumor size was measured two or three times per week using a gage, and the volume was calculated considering the tumor as an oval according to the formula:



Statistical analyses were performed with two-way repeated-measures analysis of variance (repeated measures ANOVA (RM ANOVA)).

To assay tumor cell proliferation, 100 μg/g body weight of BrdU (Cell Proliferation Kit II; Roche) was intraperitoneally injected into tumor-bearing mice that were sacrificed 2 hours later. Tumors were excised and washed with PBS before fixation in 4% paraformaldehyde (PFA) at 4°C for 24 hours, and BrdU detection was performed as previously described [13]. To examine experimental metastasis, 1.0 × 10^6 ^MDA-MB-231/sFRP1-P2 cells or control-P2 cells were injected into the tail vein of female Balb/c nude mice (5 to 6 mice per group). Fifty-three days after the injection, the mice were sacrificed, lungs were dissected and the total number of surface lung metastases was determined. For western analyses, excised tumors were snap frozen and pulverized in liquid nitrogen and lysed in SDS buffer (100 mM Tris- HCl pH 7.6, 2% SDS, 10 mM dithiothreitol, 2 mM sodium vanadate, 0.5 mM ethylenediamine tetraacetic acid) by incubation at 95°C for 10 minutes.

### Immunohistochemistry and functional vessel analysis on tumor sections

To detect functional vessels in tumors, 100 μl of 2 μg/μl solution of fluorescein-labeled *Lycopersicon esculentum *lectin (Vector Labs, Burlingame, CA, USA) was injected into tail veins of tumor-bearing mice [14], and the mice were sacrificed 5 minutes later. Tumors were excised, fixed in 4% paraformaldehyde in PBS for 48 hours at 4°C, followed by an overnight incubation in 30% sucrose in PBS at 4°C, and then embedded in tissue-Tec O.C.T. Compound 4583 (Sakura, Tokyo, Japan). Frozen sections (9 μm) were subjected to IHC analysis to detect tumor-associated vessels using rat anti-mouse CD31 (diluted 1:100; BD Pharmingen) and goat anti-rat ALEXA 568 (diluted 1:200; Molecular Probes). Staining was performed using Discovery XT (Ventana Medical Systems, Inc., Tucson, AZ, USA). Pictures were taken with a Z1 microscope (Carl Zeiss, Jena, Germany) and were analyzed with IMARIS (Bitplane, Zurich, Switzerland) to calculate the co-localized area. For detection of cyclin D1, frozen tumor sections (9 μm) were subjected to IHC using SP4 (diluted 1:100) and Biotin-SP-conjugated affinipure donkey anti-rabbit IgG (diluted 1:100). Staining was carried out using Discovery XT with sCC1 pretreatment. Pictures were taken with an Eclipse E600 (Nikon) and were analyzed with IMARIS (Bitplane) to calculate the signal intensity.

### RNA isolation, real-time PCR and microarray analyses

Cultured cells were collected when plates were 70 to 80% confluent and total RNA was extracted using the RNeasy Mini kit (Qiagen, Venlo, The Netherlands). To extract total RNA from tumors, dissected tumors were put into RNAlater (Qiagen) overnight at 4°C, followed by RNA extraction using TRIzol reagent (Invitrogen) and washing using the RNAeasy Mini kit according to the manufacturer's instructions. RNA from mammary tumors (six MDA-MB-231/sFRP1-P1 tumors and five control tumors) and cultured cells (three MDA-MB-231/sFRP1 clones and three control clones) were individually amplified and labeled using the Ambion MesageAMP III RNA Amplification Kit (Applied Biosystems, Austin, TX, USA). Biotinylated, fragmented cRNA was hybridized to Affymetrix U133 plus 2.0 human GeneChips™ (Affymetrix, Santa Clara, CA, USA).

Expression values were estimated using the GC-RMA implementation found in Genedata's Refiner 4.5 software (Genedata AG, Basel, Switzerland). Quantile normalization and median scaling were performed in order to standardize array signal distributions to facilitate the comparison between *in vitro *cultured cells and *in vivo *tumor samples. Probesets showing statistically different expression profiles (one-way analysis of variance with *P *< 0.01; Benjamini and Hochberg *Q *values determined to minimize the false discovery rate) and specific pairwise fold changes were clustered by rank correlation with *R *> 0.8 for the first criterion and *R *> 0.885 for the second criterion using the Profile Distance Search function of Genedata's Analyst 4.5 tool (Genedata AG, Basel, Switzerland). All of the microarray data are stored in Gene Expression Omnibus [GEO:GSE13806].

For the quantitative real-time PCR, each sample cDNA was made from 2.5 μg RNA using Ready-To-Go™ You-Prime First-Strand Beads (GE Healthcare). Quantitative real-time PCR was performed with ABI Prism 7000 (Applied Biosystems, Austin, TX, USA) using ABsolute SYBR Green ROX Mix (THERMO Scientific, Waltham, MA, USA) following the manufacturer's guidelines. The primer sequences used for quantitative real-time PCR are as follows: human c-Myc forward, 5'-CCTACCCTCTCAACGACAG-3'; human c-Myc reverse, 5'-CTTGTTCCTCCTCAGAGTCG-3'; human β-actin forward, 5'-TGTCCACCTTCCAGCAGATGT-3'; and human β-actin reverse, 5'-CGCAACTAAGTCATAGTCCGCC-3'.

## Results

### Ectopic sFRP1 expression in MDA-MB-231 cells blocks WNT signaling

**Figure 1:**
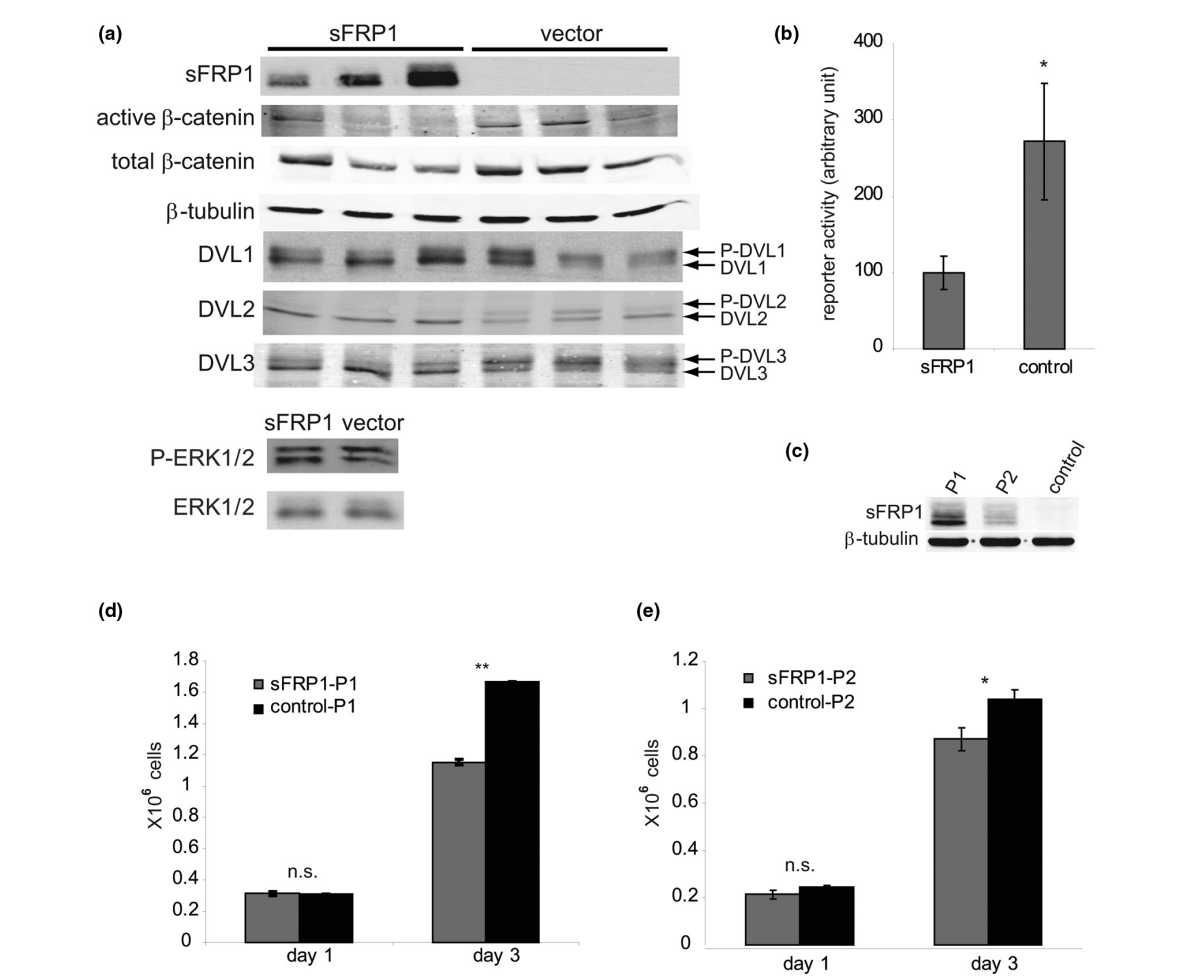
Ectopic expression of sFRP1 in MDA-MB-231 breast cancer cells. **(a) **Western analysis was performed on lysates of three MDA-MB-231/secreted Frizzled-related protein 1 (sFRP1) clones and three MDA-MB-231/control clones and the levels of active β-catenin, total β-catenin, α-tubulin and DVL1 to DVL3 determined with specific antisera. Myc-tagged sFRP1 was detected with a Myc-specific antiserum. Lower panel: level of total ERK1/2 and P-ERK1/2 in a MDA-MB-231/sFRP1 clone and a MDA-MB-231/control clone. **(b) **Activity of the WNT/β-catenin pathway was measured using the TOPFlash T-cell factor reporter system. MDA-MB-231/sFRP1-P1 and control-P1 cells were transiently co-transfected with the TOPFlash reporter plasmid and a pRL-CMV control plasmid, and the reporter activity was measured 48 hours later with a luminometer. *y *axis, TOPFlash reporter activity normalized by pRL-CMV control activity (arbitrary units), average ± standard error. *P < 0.05. **(c) **Level of Myc-tagged sFRP1 in two pools of MDA-MB-231/sFRP1 cells determined by western analysis using a Myc-specific antiserum. α-Tubulin levels served as a control. P1 is a mixture of the three clones shown in (a); P2 was generated from > 100 sFRP1-infected clones. **(d) **MDA-MB-231/sFRP1-P1 and **(e) **MDA-MB-231/sFRP1-P2 and control-P1 and control-P2 were seeded on six-well dishes (200,000 cells/well) in DMEM, 10% FCS. After 1 day and 3 days, three wells per condition were counted and the average cell numbers were calculated ± standard error of the mean. **P < 0.01, *P < 0.05, n.s. = not significant.

Interference with autocrine WNT signaling via sFRP1 has been shown to block *in vitro *proliferation of human breast cancer cell lines [6,7]. In the following experiments we examined the effects of blocking WNT signaling using the basal-like breast cancer model MDA-MB-231 [15]. Vectors encoding Myc-tagged sFRP1 and the empty control were transfected into MDA-MB-231 cells, which express no sFRP1 mRNA (data not shown), and stable clones were selected in G418-containing medium. Three MDA-MB-231/sFRP1 clones expressing moderate to strong levels of the Myc-tagged sFRP1, as well as control clones, were selected for further analyses (Figure 1a).

WNT pathway activity was examined in the cells using various markers. As a consequence of WNT binding to FZD, cytoplasmic scaffolding proteins of the Dishevelled family (DVL1, DVL2 and DVL3) become phosphorylated on serine and threonine residues. DVL phosphorylation, which is the most proximal signaling event downstream of FZD activation, can be monitored by a decrease in the electrophoretic mobility of p-DVL [16,17]. DVL1, DVL2 and DVL3, total β-catenin and stabilized active β-catenin were examined by western analysis in the individual clones. In the sFRP1-expressing cells, there was a decrease in the level of the three p-DVLs, compared with the vector control cells. Furthermore, the level of total β-catenin and active β-catenin was reduced in the two clones expressing the highest level of sFRP1 (Figure 1a).

Next we examined transcriptional activity of the canonical WNT signaling pathway following transient expression of the TOPFlash TCF reporter plasmid in the cells. TOPFlash luciferase reporter activity showed a significant 2.7-fold decrease in the sFRP1-expressing cells compared with controls (Figure 1b), confirming that ectopic expression of sFRP1 reduces canonical pathway activity in MDA-MB-231 cells. The phosphorylation status of ERK, another signaling protein that is active in MDA-MB-231 cells, was not altered in the sFRP1-expressing cells (Figure 1a), suggesting that the effects of sFRP1 are specific for the WNT pathway.

For further studies, the three sFRP1-expressing clones were pooled (P1) and a second pool of sFRP1-expressing MDA-MB-231 cells consisting of > 100 clones was generated (P2). Corresponding control pools, control-P1 and control-P2, were also generated. Quantification of a western analysis shows that MDA-MB-231/sFRP1-P1 has 2.8-fold higher levels of sFRP1 than does MDA-MB-231/sFRP1-P2 (Figure 1c).

### sFRP1 decreases proliferation of MDA-MB-231 cells

Next we examined effects of sFRP1 on tumor cell proliferation. MDA-MB-231 parental cells were inhibited by treatment with sFRP1 CM (see Additional data file 1). Furthermore, ectopic expression of sFRP1 in MDA-MB-231 cells also caused a decrease in proliferation (Figure 1d, e). One day after plating there was no significant difference between the control cells and the sFRP1-expressing cells. After 3 days, however, we observed a significant difference in the sFRP1-P1 cells and sFRP1-P2 cells compared with control cultures. This effect appears to be dependent on sFRP1 expression levels since, in comparison with controls, there is a 31% and a 16% reduction in proliferation of MDA-MB-231/sFRP1-P1 (Figure 1d) and MDA-MB-231/sFRP1-P2 (Figure 1e) cells at day 3, respectively.

### Xenografts of sFRP1-expressing MDA-MB-231 cells show reduced growth in nude mice

**Figure 2:**
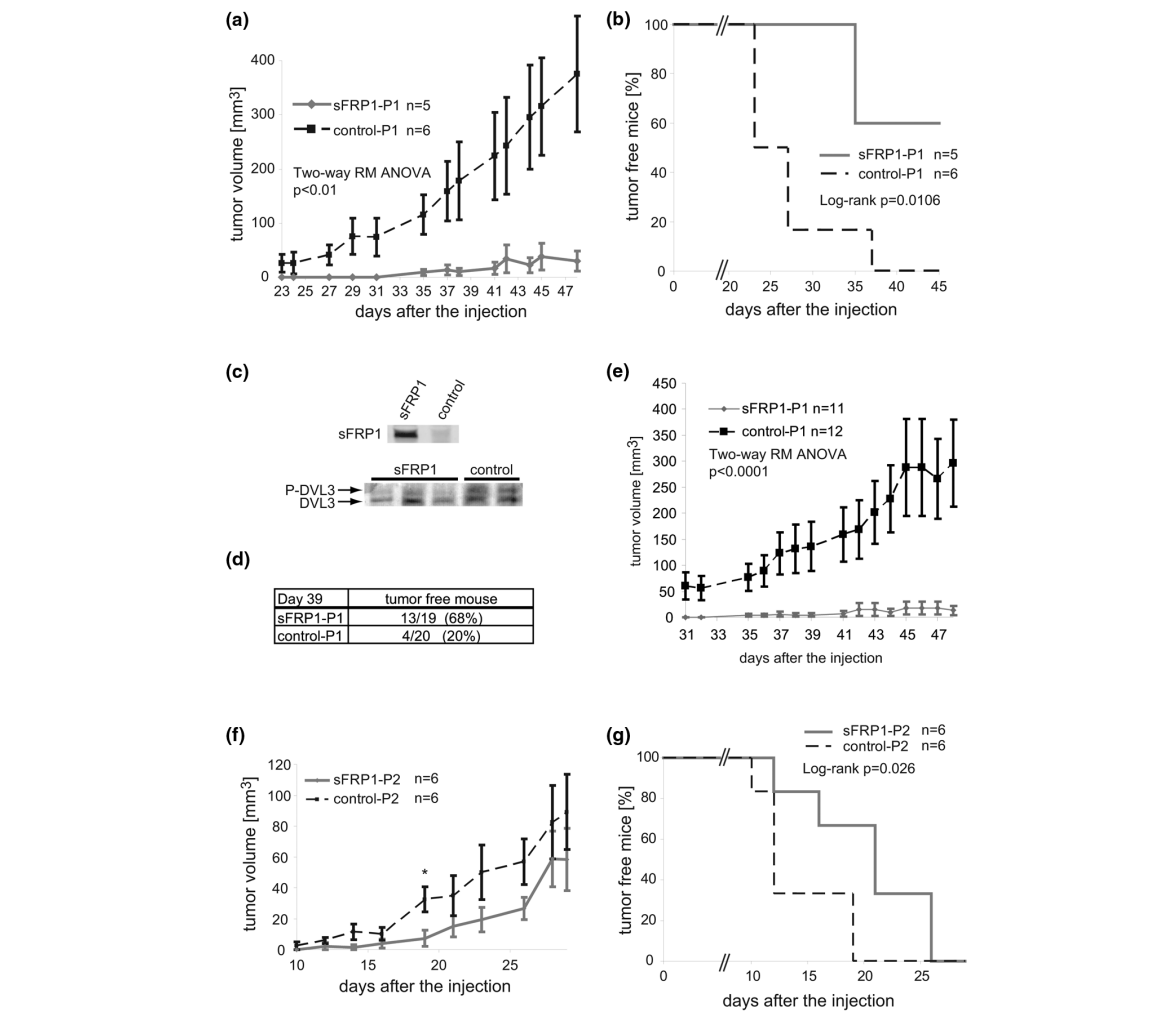
Ectopic expression of sFRP1 in MDA-MB-231 cells suppresses *in vivo *tumor formation. **(a), (b) **MDA-MB-231/secreted Frizzled-related protein 1 (sFRP1)-P1 cells and control cells (1 × 10^6^) were injected into mammary fat pads of the indicated number of Balb/c nude mice, and the tumor formation and growth were monitored: (a) average tumor volume, *P *< 0.01 (two-way repeated-measures analysis of variance (RM ANOVA)); (b) percentage of tumor-free mice on the indicated days after injection, *P *= 0.0106 (log-rank test). **(c) **Lysates prepared from individual tumors at the end of the experiment were monitored for Myc-tagged sFRP1 (upper panel) and for p-DVL3 and DVL3 (lower panel) by western analyses. **(d) **Three independent xenograft experiments using MDA-MB-231/sFRP1-P1 cells (n = 19) and control-P1 cells (n = 20) were performed and data were pooled to calculate the percentage of tumor-free mice 39 days after the injection. **(e) **Data from the indicated number of mice generated in two independent xenograft experiments with MDA-MB-231/sFRP1-P1 cells and control-P1 cells ((a) and Additional data file 2, Figure 2a) were pooled to yield the tumor growth curve, *P *< 0.0001 (two-way RM ANOVA). **(f), (g) **MDA-MB-231/sFRP1-P2 cells and control P2 cells (1 × 10^6^) were injected into mammary fat pads of the indicated number of Balb/c nude mice, and tumor formation and growth were monitored: (f) average tumor volume, *P < 0.01 on day 19 (Student's *t *test); (g) percentage of tumor-free mice on the indicated days after injection, *P *= 0.026 (log-rank test). (a), (d), (e) Tumor growth curves shown as the average tumor volume ± standard error.

To test the *in vivo *effects of sFRP1 expression, control and sFRP1-expressing MDA-MB-231 cells were injected into the mammary fat pads of female nude mice and tumor growth was monitored. There was a significant reduction in tumor outgrowth in mice injected with MDA-MB-231/sFRP1-P1 cells compared with control cells (*P *< 0.01, two-way RM ANOVA) (Figure 2a). Furthermore, the time to detection of the first tumors was much shorter after injection of MDA-MB-231/control-P1 cells, compared with MDA-MB-231/sFRP1-P1 cells (23 days vs. 35 days, respectively) (Figure 2b). Moreover, three mice injected with the MDA-MB-231/sFRP1-P1 cells remained tumor-free at day 45, when the experiment was terminated. In contrast, all of the mice injected with MDA-MB-231/control-P1 cells had tumors (Figure 2b). Western analysis carried out on tumor lysates revealed that sFRP1 was present in tumors arising from MDA-MB-231/sFRP1-P1 cells and WNT signaling was downregulated, since there was a decrease in the amount of p-DVL3 in comparison with control tumors (Figure 2c).

MDA-MB-231/sFRP1-P1 cells were tested in two additional experiments that yielded similar results (see Additional data file 2). While there was some variation in the time of tumor onset in the individual experiments, the time to appearance of the first tumor was consistently longer following injection of the MDA-MB-231/sFRP1 cells, in comparison with control cells (Figure 2b; see Additional data file 2). The data pertaining to the number of tumor-free mice were pooled for the three experiments, showing that 68% of the mice injected with MDA-MB-231/sFRP1-P1 cells were tumor-free at day 39, while only 20% of the control animals were free of tumors (Figure 2d). The data on tumor outgrowth kinetics were pooled for two experiments (Figure 2e), yielding a highly significant difference in the outgrowth ability of sFRP1-expressing cells (*P *< 0.0001, two-way RM ANOVA).

MDA-MB-231/sFRP1-P2 cells, which express 2.8-fold less sFRP1 than do the MDA-MB-231/sFRP1-P1 cells (Figure 1c), also grew more slowly than control cells following injection in the mammary gland (Figure 2f). Although the effect on tumor growth did not reach significance using two-way RM ANOVA and all animals had tumors at the end of the experiment (Figure 2g), tumor onset was delayed significantly in the cohort injected with MDA-MB-231/sFRP1-P2 cells (*P *= 0.026, log-rank test) (Figure 2g). The *in vivo *results together with the data on *in vitro *proliferation (Figure 1d, e) suggest that higher levels of sFRP1 cause a stronger blockade of WNT pathway activity, leading to a more pronounced effect on the time to tumor onset and tumor outgrowth.

### MDA-MB-231/sFRP1-expressing cells have a lower migratory ability and form fewer metastases

**Figure 3:**
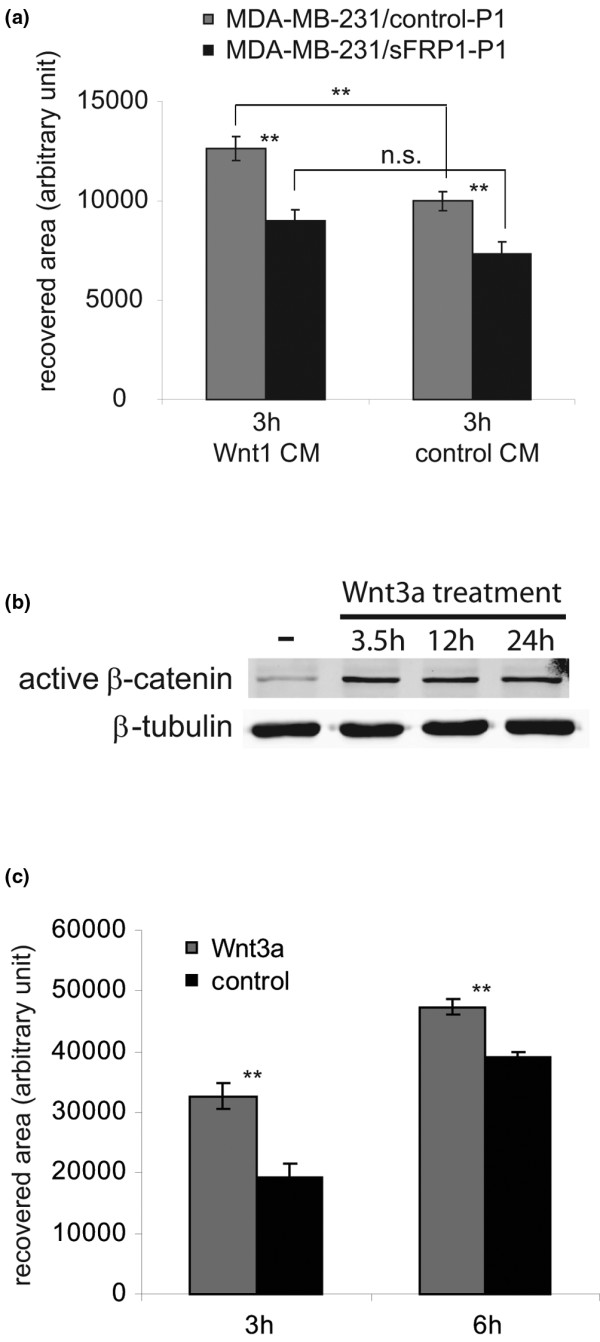
WNT promotes MDA-MB-231 cell migration. **(a) **Confluent monolayers of MDA-MB-231/secreted Frizzled-related protein 1 (sFRP1)-P1 cells and control-P1 cells were scratched, the medium was changed to Wnt1 conditioned medium (CM) or control CM, and 3 hours later the recovered areas were calculated on nine randomly chosen wound edges. Results presented in arbitrary units. Average recovered areas calculated ± standard error of the mean (SEM). **P < 0.01, n.s. = not significant (*P *> 0.05). **(b) **MDA-MB-231 cells were treated with 100 ng/ml recombinant Wnt3a for the indicated time. Active β-catenin was detected by western analysis using specific antiserum; α-tubulin level serves as a loading standard. **(c) **Confluent monolayers of MDA-MB-231 cells cultured in DMEM, 10% FCS were changed to DMEM, 10% FCS with or without 100 ng/ml recombinant Wnt3a, and 90 minutes later the monolayer surface was scratched. The recovered area was measured on nine randomly chosen wound edges, 3 and 6 hours later. *y *axis, recovered area (arbitrary unit) ± SEM. **P < 0.01.

Acquisition of migratory ability by cancer cells is an important characteristic contributing to metastatic tumor cell spread [18]. Accordingly, we also examined the effect of WNT signaling on the migratory ability of MDA-MB-231 cells, using a wound healing assay. Confluent monolayers of MDA-MB-231/sFRP1-P1 cultures and control-P1 cultures were scratched and the medium was changed to Wnt1 CM or control CM. In response to Wnt1 CM, the control MDA-MB-231 cells migrated significantly more rapidly into the wounded area compared with cultures treated with control CM (Figure 3a, gray bars). In contrast, Wnt1 treatment of MDA-MB-231/sFRP1-P1 cells did not significantly stimulate migration (Figure 3a, black bars), reflecting the ability of sFRP1 to block Wnt1-mediated FZD activation.

We also performed a migration assay using the parental MDA-MB-231 cells treated with purified recombinant Wnt3a to confirm the results obtained with Wnt1 CM (recombinant Wnt1 is not commercially available). Wnt3a stimulated the canonical pathway as shown by the increased level of active β-catenin (Figure 3b), and increased the migratory ability of the cells in a wound closure assay (Figure 3c). In summary, in MDA-MB-231 cells, activation of the WNT pathway has a positive effect on cell migration; while sFRP1 lowers the proliferative and migratory ability of the MDA-MB-231 cells.

**Figure 4:**
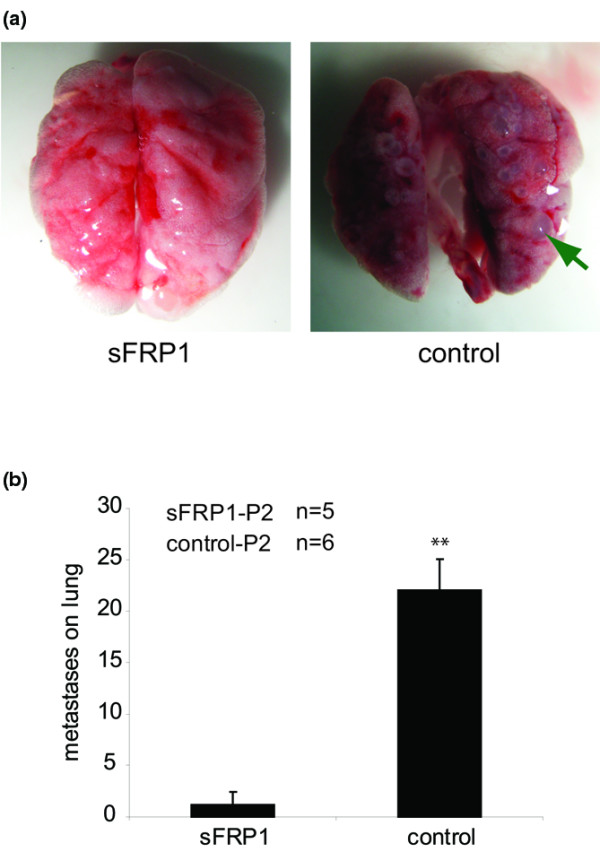
sFRP1 inhibits the metastatic potential of MDA-MB-231 tumor cells. MDA-MB-231/secreted Frizzled-related protein 1 (sFRP1)-P2 cells or control-P2 cells (1 × 10^6^) were injected into five and six Balb/c nude mice, respectively, through the tail vein; 53 days later the mice were sacrificed and the lungs were dissected. **(a) **Representative picture of lungs from the mice injected with MDA-MB-231/sFRP1-P2 cells (left) and control-P2 cells (right); arrow, metastatic lesion. **(b) **The total number of lung surface metastases was determined for all the mice and the average is shown ± standard error of the mean. **P < 0.01.

Next we tested the effect of WNT pathway blockade on the *in vivo *metastatic ability of MDA-MB-231 cells. Populations of MDA-MB-231/sFRP1 cells and control cells were injected into nude mice through the tail vein [19]; 53 days later the mice were sacrificed and the lungs were analyzed for metastatic foci. There was a dramatic difference in the number of metastatic foci arising from sFRP1-expressing cells compared with control cells. A typical lung from an animal injected with control MDA-MB-231 cells and with sFRP1-expressing cells is shown (Figure 4a). A quantitative analysis of the lungs revealed that there is a significant decrease in the number of metastases arising in mice injected with sFRP1-expressing cells in comparison with control MDA-MB-231 cells (Figure 4b). In conclusion, sFRP1-mediated blockade of WNT signaling impairs the *in vitro *migratory ability and the *in vivo *metastatic ability of MDA-MB-231 tumor cells.

### Mechanisms contributing to the *in vivo *anti-tumor effects of sFRP1

**Figure 5:**
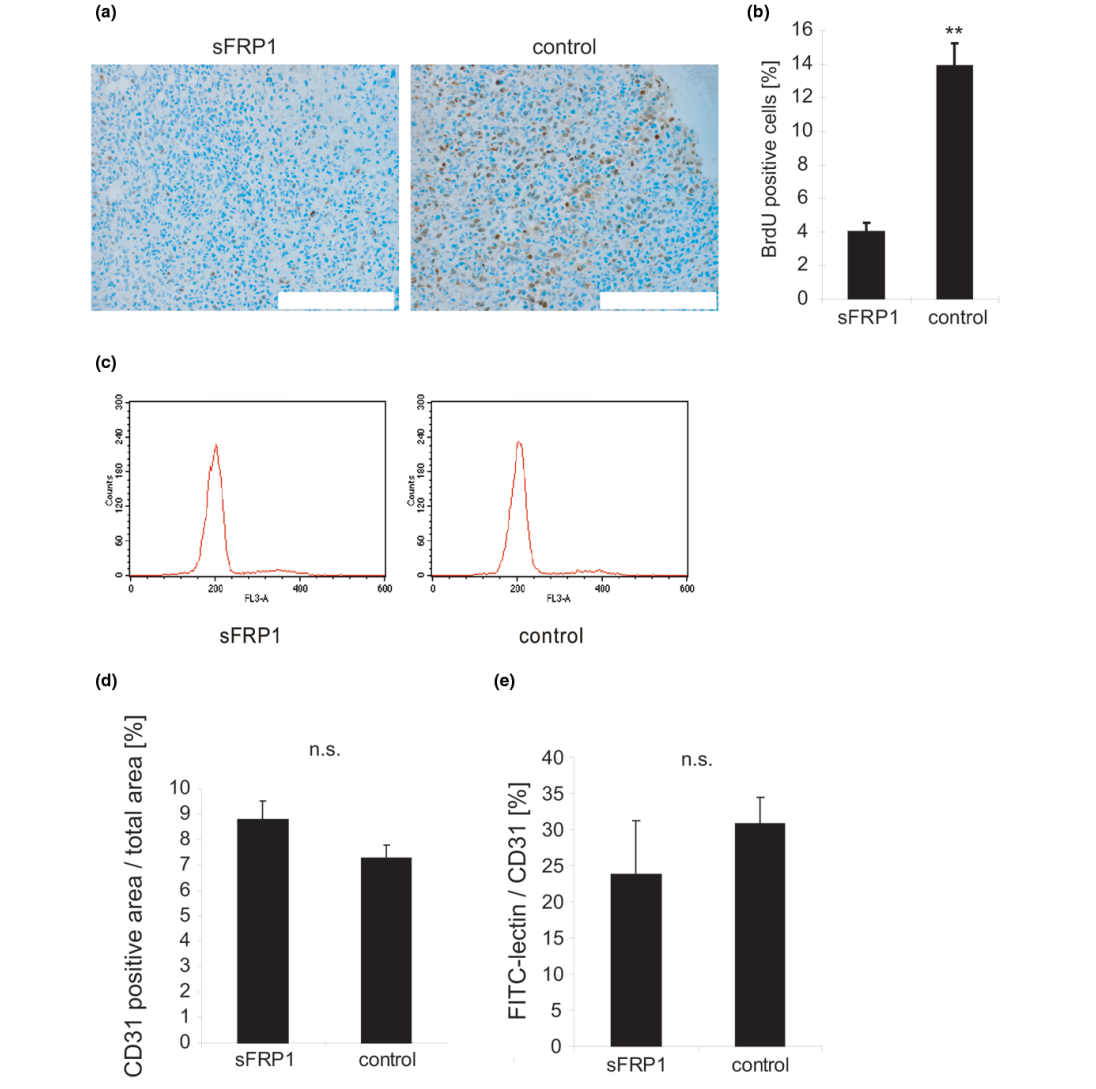
Analysis of proliferation and tumor angiogenesis in MDA-MB-231/sFRP1 xenografts. **(a) **Mice bearing MDA-MB-231/secreted Frizzled-related protein 1 (sFRP1)-P1 or control-P1 tumors were injected with bromodeoxyuridine (BrdU) and sacrificed 2 hours later. Tumors of similar weight from each group were sectioned and stained with anti-BrdU antibody. Bar = 250 μm. **(b) **BrdU-positive nuclei and total nuclei were counted in nine randomly chosen areas from each section. Bar graph shows the quantification ± standard error of the mean (SEM). **P < 0.01. **(c) **Aniokis assays were performed by seeding cells in 1% FCS-containing medium on polyHema-coated plates to prevent adhesion. Cells were harvested 24 hours later, stained with propidium iodide and analyzed with a FACScalibur. Representative cell cycle distribution of three independent control and sFRP1-expressing MDA-MB-231 clones shown. **(d) **Total blood vessels in tumor sections visualized by staining for the endothelial cell marker CD31. *y *axis, ratio of CD31^+ ^area/total area. Average calculated from five sFRP1-expressing and eight control tumors ± SEM. n.s., not significant. **(e) **Functional blood vessels in tumor-bearing mice visualized by injecting FITC-lectin into tail veins 5 minutes before sacrificing. The FITC^+ ^and CD31^+ ^areas were measured on tumor sections and the average was calculated on three sFRP1-expressing and five control tumors. *y *axis, FITC^+ ^area/CD31^+ ^area ratio ± SEM. n.s., not significant. **(d), (e) **Image analysis performed using IMARIS software (Bitplane).

Our next goal was to uncover the mechanism underlying the ability of sFRP1 to decrease the mammary tumor-forming potential of MDA-MB-231 cells. This could be tumor cell intrinsic, resulting from downregulation of WNT signaling, and/or extrinsic via secreted sFRP1 effects on tumor-associated cells; both possibilities were tested. *In vivo *tumor cell proliferation was evaluated by examining BrdU incorporation in control tumors and sFRP1-expressing tumors. Incorporated BrdU was detected with a specific antiserum (Figure 5a) and staining was quantified. There was a 70% reduction in BrdU staining in tumors arising from MDA-MB-231/sFRP1-P1 cells compared with control tumors (Figure 5b). Apoptosis, as measured by western blotting for cleaved caspase-3, was low in the MDA-MB-231 control tumor lysates and was not increased in the MDA-MB-231/sFRP1-P1 tumor lysates (data not shown). Moreover, expression of sFRP1 in MDA-MB-231 cells did not render them sensitive to anoikis-induced cell death (Figure 5c), which rules out the possibility that slower tumor outgrowth reflects lower numbers of viable cells at the time of inoculation. In summary, the results suggest that sFRP1 downregulation of WNT signaling has a strong effect on tumor cell proliferation, but not on survival.

sFRP1 has been reported to block *in vivo *neovascularization [20]. We therefore considered the possibility that the density or the functionality of the tumor-associated vessels might be impaired in sFRP1-expressing tumors. Vasculature was visualized by tail vein injection of tumor-bearing mice with FITC-labeled *L. esculentum *lectin [14] 5 minutes before the animals were sacrificed, which allows only functional vessels to be perfused. Tumor sections were prepared and the associated endothelial cells were stained for CD31, while functional vessels were visualized via the FITC signal. There was no significant difference in the total vessel area or the ratio of FITC-positive/CD31-positive vessels in sFRP1-expressing tumors compared with control tumors (Figure 5d, e), suggesting that sFRP1 does not influence the number or the functionality of tumor-associated blood vessels. In summary, these results suggest that sFRP1-mediated blockade of WNT pathway activity in tumor cells is an important factor contributing to the slower outgrowth of the MDA-MB-231/sFRP1 tumors in mammary glands.

### Identification of genes whose expression level is selectively altered *in vivo *in sFRP1-expressing tumors

To identify target genes that are controlled by sFRP1 expression and might influence proliferation of the MDA-MB-231 cells, we undertook a genome-wide transcriptome analysis using microarrays. RNA isolated from individual tumors arising after injection of MDA-MB-231/sFRP1-P1 cells and control-P1 cells, as well as RNA from *in vitro *cultured MDA-MB-231/sFRP1-P1 cells and control-P1 cells, was analyzed.

Considering data generated from six sFRP1-expressing tumors and five control tumors, there were 1,753 probesets (1,246 genes) whose signals changed more than 1.5-fold (*P *< 0.01, Student's *t *test) in the tumors arising from MDA-MB-231/sFRP1-P1 cells compared with tumors arising from control-P1 cells. The same analysis performed on *in vitro *cultured samples revealed 428 probesets (332 genes) that had a 1.5-fold difference (*P *< 0.01, Student's *t *test). Only 69 probesets (54 genes) overlapped between the two analyses, clearly showing the substantial difference in gene expression between *in vivo *tumors and *in vitro *cultured cells.

All of the microarray data were examined for characterized WNT pathway target genes. We identified 16 genes where at least one of the probesets showed a tendency for suppression in MDA-MB-231/sFRP1-expressing cells and tumors (see Additional data files 3 and 4).

### Cell cycle regulators are altered in sFPR1-expressing MDA-MB-231 xenografts

**Figure 6:**
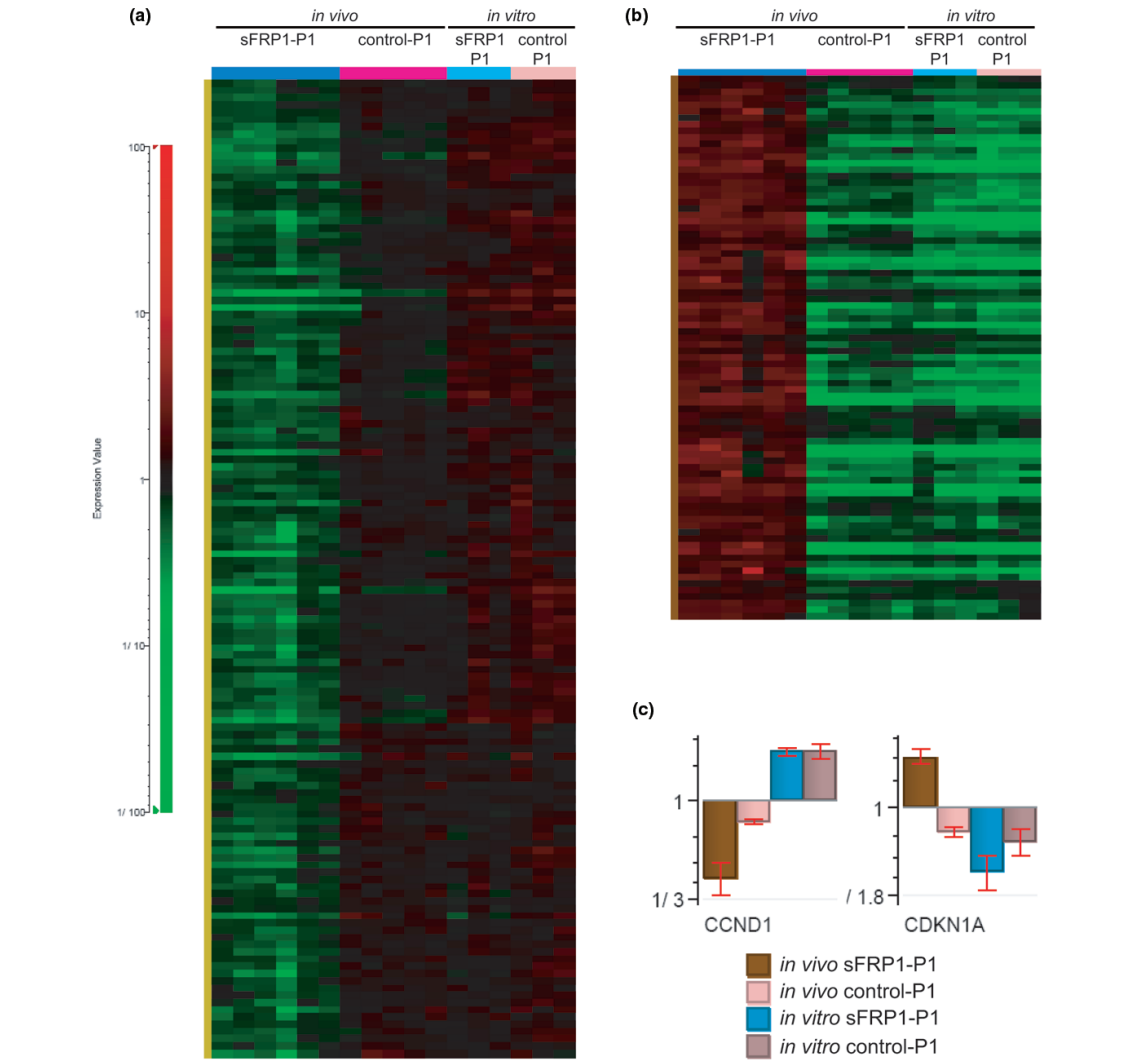
Microarray analysis comparing gene expression profiles generated from tumors and from *in vitro *cultured cells. **(a) ***y *axis, 135 probesets (106 genes) showing low expression in secreted Frizzled-related protein 1 (sFRP1)-positive tumors and moderate-high expression in control tumors, in *in vitro *cultured sFRP1-positive and in control cells. **(b) ***y *axis, 84 probesets (62 genes) showing high expression in sFRP1-positive tumors, and low expression in control tumors, in *in vitro *cultured sFRP1-positive and in control cells. (a), (b) Left to right, columns represent six sFRP1-positive tumors, five control tumors, three MDA-MB-231/sFRP1 clones and three MDA-MB-231/control clones (from Figure 1a). Genes with high, moderate and low expression are indicated in red, black and green, respectively. **(c) **Normalized gene expression of *CCND1 *and *CDKN1A *in MDA-MB-231/sFRP1-P1 and control-P1 tumors, and in MDA-MB-231/sFRP1-P1 and control P1 *in vitro *cultured cells.

*In vitro *proliferation of MDA-MB-231/sFRP1-P1 cells was decreased by 30% compared with control cultures (Figure 1), while the *in vivo *effects of WNT pathway blockade appeared to be much stronger. For example, in comparison with control MDA-MB-231 cells, outgrowth of MDA-MB-231/sFRP1-P1-generated tumors was significantly slower; and tumor-free mice remained in this cohort in each experiment (Figure 2). We therefore decided to identify transcripts that were only altered *in vivo *in MDA-MB-231/sFRP1-expressing tumors. We screened the previously mentioned 1,753 probesets that were originally identified by a comparison of the MDA-MB-231/sFRP1 tumors with control tumors (see above) using the Profile Distance Search function of Genedata's Analyst 4.5 tool. In this analysis we looked for genes whose expression was significantly altered only in tumors arising from MDA-MB-231/sFRP1-P1 cells compared with tumors arising from control-P1 cells, with cultured MDA-MB-231/sFRP1-P1 cells or with cultured control-P1 cells. This resulted in 135 probesets (106 genes) that were downregulated (Figure 6a) and 84 probesets (62 genes) that were upregulated (Figure 6b) only in the sFRP1-positive tumors (see Additional data files 5 and 6 for lists of genes and their fold change).

The microarray analysis showed that the signal from a probeset for *CCND1 *(encoding cyclin D1) was downregulated and the probeset for *CDKN1A *(gene encoding p21^Cip1^) was upregulated *in vivo*, in tumors resulting from MDA-MB-231/sFRP1-P1 cell injection (Figure 6c). The *CCND1 *promoter has a consensus lymphoid enhancer binding factor-1 binding site [21], and in some cancer models its expression is controlled by β-catenin/TCF activation [22]. Our results suggest that *CCND1 *might also be a direct β-catenin/TCF target in MDA-MB-231 cells.

**Figure 7:**
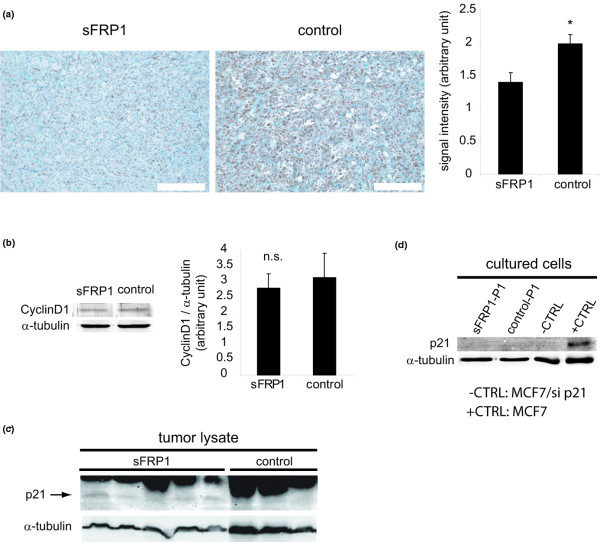
Detection of cyclin D1 and p21^Cip1 ^in tumors and in cultured cells. **(a) **Tumor sections from mice bearing MDA-MB-231/secreted Frizzled-related protein 1 (sFRP1)-P1 and control tumors were stained with cyclin D1-specific antiserum. Signal intensities, shown in the bar graph, reflect data collected from six MDA-MB-231/sFRP1-P1 tumors and eight control tumors ± standard error of the mean (SEM). *P < 0.05. **(b) **Cyclin D1 detected in lysates from *in vitro *cultured MDA-MB-231/sFRP1-P1 cells and control cells by immunoblotting. Bar graph: levels were quantified from three MDA-MB-231/sFRP1 and three control clones (from Figure 1a). Average ± SEM, n.s. = not significant. **(c) **p21^Cip1 ^detected in tumor lysates from five MDA-MB-231/sFRP1-P1 tumors and three control tumors. **(d) **Lysates from *in vitro *cultured MDA-MB-231/sFRP1-P1 and control-P1 cells had no detectable p21^Cip1^. Lysates from MCF7 control and siRNA-mediated p21^Cip1 ^knockdown tumor cells served as positive and negative controls, respectively; α-tubulin served as loading control.

Both of these genes were analyzed further based on their known roles in cell cycle regulation and proliferation. Cyclin D1 was examined by IHC in tumor sections using a specific antiserum. Quantification of the staining showed a 30% decrease in cyclin D1 in sFRP1-expressing tumors compared with control tumors (Figure 7a). Western analysis for cyclin D1, carried out on lysates prepared from MDA-MB-231/sFRP1-P1 cultures and control cultures, revealed no significant difference in expression (Figure 7b). Western analysis revealed that p21^Cip1 ^was present in tumors resulting from injection of MDA-MB-231/sFRP1-P1 cells, while none was detectable in control tumors (Figure 7c). Considering the *in vitro *cultured cells, neither MDA-MB-231/sFRP1-P1 cells nor control-P1 cells had detectable levels of p21^Cip1 ^protein (Figure 7d). As predicted from the array data (Figure 6c), therefore, the decrease in cyclin D1 and the increase in p21^Cip1 ^are observed *in vivo *in the sFRP1-expressing tumors.

Considering that c-Myc is a WNT pathway target [23,24] that regulates cyclin D1 [22] and p21^Cip1 ^expression [25], we also examined c-Myc. There were no significant changes in c-Myc RNA levels in sFRP1-expressing tumors; however, c-Myc protein levels were lower in all of the sFRP1-expressing tumors compared with the control tumors (see Additional data file 7). In summary, the *in silico *analysis carried out on RNA of tumors arising from the MDA-MB-231/sFRP1 cells revealed altered levels of target genes (*CCND1 *and *CDKN1A*) that probably contribute to the anti-proliferative effects of sFRP1 expression. Moreover, our results also show the strong influence that the *in vivo *tumor environment has, not only on gene expression, but also on c-Myc protein. Decreased c-Myc levels might also contribute to the *in vivo *activity of sFRP1.

## Discussion

Aberrant activation of WNT signaling plays an important role in many types of human cancer, warranting therapeutic approaches to target the pathway [26]. Wnt1 was the first identified oncogene activated by mouse mammary tumor virus insertional mutagenesis [27], establishing the potential of aberrant WNT expression to promote mammary cancer. Currently, it is well documented that multiple WNT ligands and FZD receptors are expressed in primary human breast tumors and breast cancer cell lines [3,7-9], making it difficult to identify an individual ligand/receptor complex that could serve as a cancer target. Using broad antagonists – including the cysteine-rich domain of the FZD8 receptor [28] or sFRP1 [6,7,29] – to interfere with WNT/FZD binding, however, the potential of targeting WNT binding to FZD as a therapeutic approach in breast cancer [6,7] and in other cancers [28,30] has been demonstrated.

Aberrant methylation of the sFRP1 promoter is one of the most consistent alterations in human cancer. In addition to breast tumors that have low sFRP1 levels [10-12,29,31], sFRP1 suppression has been described in colon tumors [32], ovarian tumors [33], bladder tumors [34] and prostate tumors [35]. Based on its widespread loss, interest in testing the effects of sFRP1 treatment in tumor models has been high. Indeed, sFRP1 has also been shown to impact on transforming properties of breast cancer cells [6,29] and cervix cancer cells [36]; while sFRP2 has been shown to block proliferation of gastric cancer cells [37]. We have previously shown that proliferation of the estrogen-receptor-positive MCF7 and T47D, and the ErbB2-overexpressing JIMT-1, SKBR3 and BT474 breast tumor cell lines is decreased following treatment with sFRP1 [7]. In the current study we tested the impact of ectopic sFRP1 expression in the aggressive, basal-like [15] MDA-MB-231 breast tumor cells. The results presented show that ectopic sFRP1 expression in MDA-MB-231 tumor cells blocks the migratory ability and the proliferative potential of the tumor cells, both *in vitro *and *in vivo*, supporting the proposal that blockade of WNT signaling with sFRP1 might be a general approach to target not only breast, but also other types of cancer.

In addition to testing sFRP1, we also examined the effects of specific Wnt ligands on motility and found that the canonical ligands Wnt1 and Wnt3a stimulate MDA-MB-231 cells in a wound closure assay. These results correlate well with the stimulatory effects of both Wnt ligands in other cellular models [38,39]. The Wnt/PCP pathway is considered the major mediator of cell motility. Indeed, this pathway stimulates many cytoskeleton regulators, including Rho family GTPases and Rho kinase [40]. Both Wnt1 and Wnt3a have been shown to activate RhoA [38,39], whereas the non-canonical Wnt5a promotes melanoma migration via RhoB [41]. Furthermore, Rho kinase inhibition has been shown to block the effects of Wnt3a [39]. We have also observed that the Rho kinase inhibitor Y27632 blocks Wnt3a-induced MDA-MB-231 wound closure (data not shown). In contrast to the positive effects of Wnt ligands on motility, we show here that sFRP1-mediated blockade of endogenous WNT signaling not only reduced the basal motility of the MDA-MB-231 cells, but also impaired the ability of the cells to respond to Wnt1 in a wound closure assay. sFRP1 has also been shown to block motility [39] and invasion [36] of other types of tumor cells. Importantly, the negative impact of sFRP1 on MDA-MB231 motility translated, *in vivo*, to a block in the metastatic potential of these aggressive breast tumor cells. In comparison with control MDA-MB-231 cells, we observed a 20-fold decrease in the number of lung metastasis arising from sFRP1-expressing MDA-MB-231 cells.

MDA-MB-231/sFRP1 cells also proliferated more slowly than control cells; however, the effect of sFRP1 was more striking *in vivo *than *in vitro*. Following injection of MDA-MB-231/sFRP1 cells into mammary glands of nude mice, the time to appearance of the tumors was consistently longer than that observed with control MDA-MB-231 cells. Furthermore, tumors generated by the sFRP1-expressing cells not only grew more slowly than control tumors, but there were threefold more tumor-free mice at the end of each experiment in this group. Since sFRP1 is a secreted protein, it could act extrinsically on cells in the tumor environment. We concentrated in particular on tumor-associated vessels based on the reported ability of sFPR1 to block *in vivo *neovascularization [20]. Neither the vessel number nor their functionality differed, however, in tumors generated by sFRP1-expressing cells in comparison with those of control MDA-MB-231 cells. The impact of sFRP1 on WNT signaling and downstream transcription in the MDA-MB-231 cells might therefore more probably explain the protein's strong *in vivo *effects. Indeed, there were 3.7-fold more genes whose transcription was altered by sFRP1 expression *in vivo *compared with *in vitro *(1,246 vs. 332). Furthermore, only 54 genes overlapped in the two lists. Taken together, these results demonstrate the strong impact of tumor environment on gene expression.

We also performed an in-depth analysis to identify genes that were only affected *in vivo*, in the sFRP1-expressing tumors, with the intention of finding potential targets that could account for the strong effect of sFRP1 on MDA-MB-231 tumor-forming potential. There were a total of 168 genes (106 downregulated genes and 62 upregulated genes) that were only affected *in vivo *in sFRP1-expressing tumors (see Additional data files 5 and 6). Changes in expression of two *in vivo *identified genes, *CCND1 *and *CDKN1A*, encoding important cell cycle regulators were confirmed by IHC and immunoblotting on tumor cells. Cyclin D1 and p21^Cip1 ^were found to be downregulated and upregulated, respectively, only in sFRP1-expressing tumors, which might be one reason why the impact of sFRP1 expression is stronger *in vivo*. These results raise the question as to why *CCND1 *and *CDKN1A *were only affected *in vivo*. While we can only speculate at this time, two explanations are worth discussion. First, c-Myc, which controls expression of both genes, was only downregulated in the sFRP1-expressing tumors; second, in the tumors there were major changes in expression of probesets for extracellular matrix proteins.

*MYC *is WNT pathway target [23,24]. We did not detect changes in *MYC *expression, however, either in the microarrays (see Additional data files 5 and 6) or by quantitative real-time PCR (see Additional data file 7). Interestingly, c-Myc protein was low in all of the sFRP1-expressing tumors (see Additional data file 7). C-Myc is subject to control at many levels [42,43] and the fact that c-Myc protein only changed in the sFRP-1-expressing tumors could be a reflection of the *in vivo *environment. Irrespective of the mechanism underlying these results, the fact that c-Myc stimulates cyclin D1 expression [22] and is a repressor for p21^Cip1 ^[25] suggests that lower c-Myc levels could contribute to the altered expression of both cell cycle regulators in sFRP1-expressing tumors.

Turning to the extracellular matrix components, probesets for fibronectin, laminins and collagens were found to be strongly altered (see Additional data file 8, 29 probesets for the extracellular matrix). As expected, *FN1 *– a WNT pathway target (see Additional data files 3 and 4) – was decreased in sFRP1-expressing cells and tumors. The most striking difference, however, was seen when comparing the signal of the 29 probesets in cultured cells versus tumors. On the one hand, 25 out of the 29 probesets (see Additional data file 8) were strongly increased *in vivo *in control tumors, showing the impact of the tumor environment on expression of the encoded genes. Moreover, 21 out of these 25 were downregulated in sFRP1-expressing tumors.

Fibronectin, laminin and collagen are ligands for β_1_-integrin, which was also suppressed in sFRP1-expressing cells (see Additional data file 8). The decrease in β_1_-integrin RNA was confirmed at the protein level by performing a fluorescence-activated cell sorting analysis on intact cells with an antibody that recognizes active β_1_-integrin (see Additional data file 9). Integrin engagement is therefore likely to be decreased in sFRP1-expressing MDA-MB-231 cells, which in turn is likely to influence their proliferation. On the one hand fibronectin has been shown to stimulate proliferation of cancer cells, and this was associated with increased cyclin D1 and decreased p21^Cip1 ^levels [44]. Moreover, we have previously shown that siRNA-mediated knockdown of β_1_-integrin in MDA-MB-231 cells increases p21^Cip1 ^expression and leads to a proliferative decrease [13]. We propose that integrin engagement would be more strongly affected *in vivo *since not only the receptor but also many of its extracellular matrix binding partners are decreased *in vivo*.

In summary, the results presented here show that sFRP1-mediated blockade of WNT signaling in MDA-MB-231 breast cancer cells has an impact on the *in vitro *proliferation and motility of the cancer cells. The *in vivo *effects of WNT pathway blockade were even more dramatic since we observed a strong decrease in the mammary tumor-forming potential and an impairment of lung metastases. In summary, blockade of the WNT-FZ interaction using sFRP1 has a strong effect on breast tumor growth.

## Conclusions

The results presented in this paper showing that sFRP1-mediated WNT pathway blockade strongly blocks the *in vivo *tumor forming potential of MDA-MB-231 breast cancer cells suggest that interference with WNT signaling at the ligand-receptor level may be a valid therapeutic approach in breast cancer.

## Abbreviations

BrdU: bromodeoxyuridine; CM: conditioned medium; DMEM: Dulbecco's modified Eagle's medium; DVL: Dishevelled; ERK1/2: extracellular signal-regulated kinase 1/2; FCS: fetal calf serum; FITC: fluorescein isothiocyanate; FZD: Frizzled; HRP: horseradish peroxidase; IHC: immunohistochemistry; LRP: low-density lipoprotein receptor-related protein; PBS: phosphate-buffered saline; PCR: polymerase chain reaction; RM ANOVA: repeated-measures analysis of variance; sFRP1: secreted Frizzled-related protein 1; siRNA: small interfering RNA; TCF: T-cell factor; WNT: wingless and integration site growth factor.

## Competing interests

The authors declare that they have no competing interests.

## Authors' contributions

YM planned and performed the experiments and participated in writing the paper. TS planned and discussed the experiments and performed the experiment shown in the supplemental figure in Additional data file 1. EJO performed the array and data analyses. AB performed the experiment shown in Figure 5c. NEH planned and discussed the experiments and wrote the paper.

## Supplementary Material

Additional data file 1 Adobe file containing a figure showing proliferation suppression of MDA-MB-231 cells by sFRP1 CM treatment. One thousand MDA-MB-231 cells were seeded per well of a 96-well plate and proliferation was measured with a YOPRO assay after 3 days of treatment with sFRP1 CM or control CM. Click here for file

Additional data file 2 Adobe file containing a figure showing additional xenograft experiments with MDA-MB-231/sFRP1-P1 and control P1 cells. **(a), (b) **The onset of tumor appearance in two independent xenograft experiments with MDA-MB-231/sFRP1-P1 and control-P1 cells: (a) six mice per group, *P*: 0.0179 (log-rank test); (b) eight mice per group, *P*: 0.0223 (log-rank test). Click here for file

Additional data file 3 Adobe file containing a figure showing known WNT target genes whose expression was downregulated upon sFRP1 expression both *in vitro *and *in vivo*. Normalized microarray results of established WNT pathway target genes listed in Additional data file 4. Click here for file

Additional data file 4 Word file containing a table that lists established WNT pathway target genes whose expression was suppressed upon sFRP1 expression both *in vitro *and *in vivo*. Expression of established WNT pathway target genes was examined by microarray analysis. Listed here are genes whose expression was suppressed upon sFRP1 expression both *in vitro *and *in vivo*. Click here for file

Additional data file 5 Word file containing a table that lists genes whose expression was downregulated upon sFRP1 expression only *in vivo*. List of 106 identified genes shown in Figure 6a. Fold-change refers to expression changes between sFRP1-positive and control tumors. For some genes there are multiple probesets with different values of fold-change. Click here for file

Additional data file 6 Word file containing a table that lists genes whose expression was upregulated upon sFRP1 expression only *in vivo*. List of 62 identified genes shown in Figure 6b. Fold-change refers to expression changes between sFRP1-positive and control tumors. For some genes there are multiple probesets with different values of fold-change. Click here for file

Additional data file 7 Adobe file containing a figure showing c-Myc expression in MDA-MB-231/sFRP1-P1 and control-P1 tumors. **(a) **Quantitative real-time PCR analysis for c-Myc RNA in tumor lysates from six sFRP1-positive tumors and five control tumors. Average ± standard error of the mean. n.s.: not significant. **(b) **Lysates from six MDA-MB-231/sFRP1-P1 tumors and two control-P1 tumors were examined for c-Myc and Myc-tagged sFRP1 with a c-Myc antiserum; α-tubulin serves as a loading control. Click here for file

Additional data file 8 Adobe file containing a containing a figure showing *in vitro *and *in vivo *gene expression of β_1_-integrin and extracellular matrix components. Microarray results of the genes coding β_1_-integrin and some extracellular matrix components. Click here for file

Additional data file 9 Adobe file containing a figure showing fluorescence-activated cell sorting analysis for active β_1_-integrin. MDA-MB-231/sFRP1-P1 and control-P1 cells were incubated with an antibody recognizing active β_1_-integrin, followed by incubation with FITC conjugated secondary antibody. Fluorescence was measured using a FACSCalibur machine and the percentage of gated cells stained with active β_1_-integrin was calculated. Click here for file
