# Supplementary material for: WNT signaling enhances breast cancer cell motility and blockade of the WNT pathway by sFRP1 suppresses MDA-MB-231 xenograft growth
Source: Breast Cancer Res. 2009 May 27;11(3):R32. doi: 10.1186/bcr2317 (PMC2716500; doi:10.1186/bcr2317)
Supplement: Additional data file 4 — Word file containing a table that lists established WNT pathway target genes whose expression was suppressed upon sFRP1 expression both in vitro and in vivo. Expression of established WNT pathway target genes was examined by microarray analysis. Listed here are genes whose expression was suppressed upon sFRP1 expression both in vitro and in vivo. [file bcr2317-S4.doc]

**Additional data file 4**

| NRCAM |
| --- |
| PTGS2 (COX2) |
| PPARD |
| RUNX2 |
| FZD7 (Frizzled7) |
| LEF1 |
| CCND1 (CyclinD1) |
| EDN1 (Endothelin1) |
| CD44 |
| RHOU (Ras homolog gene family, member U) |
| AXIN2 |
| JUN |
| STRA6 |
| PLAUR (Plasminogen activator) |
| TCF4 |
| FN1 (Fibronectin1) |
