# Supplementary material for: WNT signaling enhances breast cancer cell motility and blockade of the WNT pathway by sFRP1 suppresses MDA-MB-231 xenograft growth
Source: Breast Cancer Res. 2009 May 27;11(3):R32. doi: 10.1186/bcr2317 (PMC2716500; doi:10.1186/bcr2317)
Supplement: Additional data file 5 — Word file containing a table that lists genes whose expression was downregulated upon sFRP1 expression only in vivo. List of 106 identified genes shown in Figure 6a. Fold-change refers to expression changes between sFRP1-positive and control tumors. For some genes there are multiple probesets with different values of fold-change. [file bcr2317-S5.doc]

**Additional data file 5**

| Gene | fold down-regulation upon sFRP1 expression |
| --- | --- |
| FGF5 | 4.09 |
| BDNF | 4.03 |
| FBXO25 | 3.84 |
| BDNF | 3.30 |
| FGF5 | 3.28 |
| DEAF1 | 2.97 |
| DLC1 | 2.96 |
| DKFZp313A2432 | 2.77 |
| APEH | 2.70 |
| ZNF289 | 2.69 |
| FGF5 | 2.65 |
| TEX261 | 2.65 |
| GALNT10 | 2.65 |
| SLC39A4 | 2.60 |
| ARHGEF10 | 2.60 |
| TSPAN4 | 2.58 |
| LYPD6 | 2.54 |
| EPS8L2 | 2.45 |
| MFHAS1 | 2.40 |
| KLF11 | 2.40 |
| FXC1 | 2.38 |
| PTPMT1 | 2.30 |
| C9orf140 | 2.30 |
| MFHAS1 | 2.26 |
| TOLLIP | 2.22 |
| PTPN18 | 2.21 |
| FLJ11236 | 2.20 |
| EIF2B4 | 2.20 |
| ANTXR1 | 2.19 |
| MAPKAPK3 | 2.17 |
| HOMER3 | 2.16 |
| TOLLIP | 2.16 |
| MAPKAPK3 | 2.14 |
| KIAA0652 | 2.09 |
| HOMER3 | 2.09 |
| TGOLN2 | 2.08 |
| CUGBP2 | 2.07 |
| LOC283267 | 2.04 |
| RNF141 | 2.04 |
| MAGEA3 | 2.03 |
| SCD | 2.03 |
| COMTD1 | 2.02 |
| CCL28 | 1.99 |
| FLJ13305 | 1.99 |
| GSTM4 | 1.98 |
| MAGEA6 | 1.97 |
| POLE4 | 1.96 |
| RNH1 | 1.95 |
| TSTA3 | 1.95 |
| POLE4 | 1.95 |
| GRK6 | 1.94 |
| KBTBD4 | 1.94 |
| TGOLN2 | 1.94 |
| NUP98 | 1.93 |
| NAP1L4 | 1.93 |
| NPAS2 | 1.92 |
| SUSD1 | 1.90 |
| THUMPD3 | 1.88 |
| NAP1L4 | 1.85 |
| MCART1 | 1.85 |
| BOK | 1.85 |
| RIC8A | 1.85 |
| TALDO1 | 1.84 |
| MTA3 | 1.84 |
| B3GNT2 | 1.83 |
| B3GNT2 | 1.83 |
| MPHOSPH10 | 1.83 |
| SGK3 | 1.83 |
| FAM125A | 1.83 |
| CRIM1 | 1.82 |
| EIF2B4 | 1.81 |
| HRAS | 1.80 |
| PDLIM5 | 1.80 |
| AP2A2 | 1.80 |
| PEX13 | 1.80 |
| ALKBH3 | 1.79 |
| ARHGAP1 | 1.78 |
| SERTAD2 | 1.76 |
| C19orf12 | 1.76 |
| STOML1 | 1.76 |
| RAP2B | 1.74 |
| ASB1 | 1.74 |
| SMEK2 | 1.74 |
| LEPROTL1 | 1.73 |
| SAAL1 | 1.73 |
| PSMD13 | 1.70 |
| C6orf108 | 1.69 |
| CCDC75 | 1.67 |
| ASH2L | 1.65 |
| ING5 : LOC727773 | 1.65 |
| MOBKL1B | 1.64 |
| SUMO3 | 1.64 |
| IQGAP1 | 1.63 |
| PI4K2B | 1.63 |
| LOC728944 : THAP4 | 1.62 |
| ALDH3A2 | 1.62 |
| PSME4 | 1.62 |
| RAB1A | 1.62 |
| RFXANK | 1.61 |
| AKT3 | 1.61 |
| RNASEH1 | 1.60 |
| POLR2L | 1.60 |
| C9orf142 | 1.60 |
| MRPL23 | 1.59 |
| AKTIP | 1.59 |
| SETMAR | 1.58 |
| C11orf17 | 1.57 |
| SGCB | 1.57 |
| ANAPC1 | 1.57 |
| MOBKL1B | 1.57 |
| SLC4A1AP | 1.56 |
| KIAA0652 | 1.55 |
| TMEM9B | 1.55 |
| MRPL53 | 1.55 |
| C5orf30 | 1.54 |
| C2orf4P : MEMO1 | 1.54 |
| PSMC3 | 1.53 |
| AUP1 | 1.52 |
| SMEK2 | 1.51 |
| **CCND1** | **1.51** |
| CENPA | 1.51 |
