# Supplementary material for: WNT signaling enhances breast cancer cell motility and blockade of the WNT pathway by sFRP1 suppresses MDA-MB-231 xenograft growth
Source: Breast Cancer Res. 2009 May 27;11(3):R32. doi: 10.1186/bcr2317 (PMC2716500; doi:10.1186/bcr2317)
Supplement: Additional data file 6 — Word file containing a table that lists genes whose expression was upregulated upon sFRP1 expression only in vivo. List of 62 identified genes shown in Figure 6b. Fold-change refers to expression changes between sFRP1-positive and control tumors. For some genes there are multiple probesets with different values of fold-change. [file bcr2317-S6.doc]

**Additional data file 6**

| Gene | fold up-regulation upon sFRP1 expression |
| --- | --- |
| SFRP1 | 51.13 |
| PAEP | 30.29 |
| PRSS1 : PRSS2 : PRSS3 : TRY6 | 29.23 |
| SERPINA3 | 27.59 |
| ECSM2 | 26.93 |
| TRY6 | 26.15 |
| PRSS1 | 25.21 |
| ECSM2 | 23.03 |
| SERPINA1 | 20.26 |
| SLC2A10 | 19.62 |
| OBP2A : OBP2B | 12.72 |
| SERPINA1 | 11.04 |
| LOC149773 | 8.94 |
| OAS2 | 8.12 |
| GPR87 | 7.35 |
| TMEM178 | 6.76 |
| FOLR1 | 6.16 |
| LTBP2 | 6.06 |
| TIE1 | 5.81 |
| WFDC10B | 5.74 |
| APCDD1L | 5.26 |
| RARRES3 | 5.25 |
| CLU | 5.24 |
| METTL7A | 5.15 |
| CTSS | 5.11 |
| DTX3 | 5.07 |
| AIM2 | 4.97 |
| OBP2A | 4.91 |
| TMPRSS2 | 4.85 |
| BHLHB3 | 4.82 |
| GLDN | 4.80 |
| TAGLN3 | 4.72 |
| CLU | 4.62 |
| LOC286299 | 4.40 |
| HOXB3 | 4.24 |
| CASP1 | 4.24 |
| CLU | 3.99 |
| APOBEC3F : APOBEC3G | 3.91 |
| IGFBP6 | 3.84 |
| SSPN | 3.77 |
| HERC6 | 3.72 |
| FAM125A | 3.70 |
| ANK3 | 3.66 |
| LTBP2 | 3.57 |
| CTSS | 3.47 |
| CASP1 : COP1 | 3.20 |
| FLJ20035 | 3.07 |
| KLHL3 | 3.03 |
| CYP2E1 | 3.02 |
| SULT1A3 : SULT1A4 | 3.02 |
| CDRT4 | 3.00 |
| ABCC3 | 2.84 |
| USP52 | 2.75 |
| PER1 | 2.72 |
| SLC39A11 | 2.71 |
| ITGA5 | 2.56 |
| C17orf60 | 2.43 |
| FXYD5 | 2.07 |
| TAPBPL | 2.02 |
| ARHGAP26 | 1.98 |
| FXYD5 | 1.98 |
| KRT79 | 1.96 |
| PRIC285 | 1.90 |
| TIAM1 | 1.86 |
| PECAM1 | 1.77 |
| MMD | 1.75 |
| CPD | 1.70 |
| **CDKN1A** | **1.66** |
| B4GALT5 | 1.64 |
| CPD | 1.64 |
